# Ginsenoside Compound K Differentially Regulates Neuronal and Astrocytic Ca^2+^ Homeostasis Under Trimethyltin-Induced Stress

**DOI:** 10.3390/ph19091376

**Published:** 2026-08-31

**Authors:** Hayeong Jeon, Yoo Jin Kim, Geun Hee Seol

**Affiliations:** 1 Department of Basic Nursing Science, College of Nursing, Korea University, Seoul 02841, Republic of Korea; 2 BK21 FOUR Program of Transdisciplinary Major in Learning Health Systems, Graduate School, Korea University, Seoul 02841, Republic of Korea

**Keywords:** ginsenoside compound K, store-operated calcium entry, calcium homeostasis, neurodegeneration, trimethyltin chloride, neurovascular unit

## Abstract

**Background**: Intracellular Ca^2+^ dysregulation and neurovascular dysfunction are increasingly recognized as important mechanisms underlying neurodegenerative disorders, including Alzheimer’s disease (AD). Restoration of pathological Ca^2+^ imbalance has therefore emerged as a potential therapeutic strategy. Although ginsenoside compound K (CK) has demonstrated neuroprotective and anti-inflammatory properties, its role in Ca^2+^ homeostasis remains unclear. **Methods**: SH-SY5Y neuronal cells, U373 astrocytes, BV2 microglia, bEND brain endothelial cells, and MOVAS vascular smooth muscle cells were exposed to trimethyltin chloride (TMT, 5 μM, 24 h). Cell viability, real-time cell confluence, intracellular Ca^2+^ influx, and endoplasmic reticulum (ER) Ca^2+^ store release were evaluated. Ca^2+^ dynamics were analyzed using Fura-2 fluorescence, and signaling associated with CK-mediated Ca^2+^ regulation was investigated using pharmacological inhibitors. Orai1 protein expression was evaluated by Western blot analysis in SH-SY5Y and U373 cells. **Results**: TMT increased store-operated Ca^2+^ entry (SOCE)-mediated Ca^2+^ influx in SH-SY5Y neuronal cells without significant cytotoxicity. In contrast, TMT reduced both Ca^2+^ influx and cell viability in U373 astrocytes, BV2 microglia, and MOVAS cells, whereas bEND cells showed minimal changes. CK restored abnormal Ca^2+^ responses in a cell type-specific manner, reducing elevated Ca^2+^ influx in neuronal cells while restoring suppressed Ca^2+^ signaling in astrocytes. Consistent with these functional findings, Orai1 protein expression was increased by TMT in SH-SY5Y cells and attenuated by CK, whereas TMT reduced Orai1 expression in U373 cells, which was restored by CK. Pharmacological analyses suggested involvement of PKA- and LTCC-associated signaling in neuronal cells and PLC- and PLD-associated signaling in astrocytes. In both cell types, CK-associated Ca^2+^ responses were sensitive to NAC and the SOCE inhibitor BTP2, supporting ROS- and SOCE-associated mechanisms. Additional controls showed no significant inhibitor effects under control or CK-only conditions, strengthening the pharmacological interpretation of responses under TMT-containing conditions. **Conclusions**: TMT-induced Ca^2+^ dysregulation differed markedly by cell type. CK restored disrupted Ca^2+^ homeostasis in association with distinct pharmacological signaling profiles in neuronal and astrocytic cells. These findings suggest that CK may function as a cell type-specific Ca^2+^ homeostatic regulator under neurotoxic stress conditions and highlight the importance of differential Ca^2+^ regulation in neurodegenerative disease models.

## 1. Introduction

Alzheimer’s disease (AD), a progressive neurodegenerative disorder, remains a major unmet clinical challenge despite continued expansion of the therapeutic pipeline and recent advances in anti-amyloid therapies [1]. Intracellular Ca^2+^ dysregulation has emerged as a central mechanism contributing to synaptic dysfunction, neuroinflammation, mitochondrial impairment, and cognitive decline in AD [2]. Importantly, pathological Ca^2+^ dysregulation is not restricted to neurons but also involves dysfunction of the neurovascular unit (NVU), which contributes to progressive neurodegeneration [3]. Therefore, investigation of Ca^2+^ homeostasis across neuronal and vascular-related cell types may provide broader insight into AD-associated pathophysiology.

Trimethyltin chloride (TMT), a tri-substituted organotin neurotoxicant, is widely used as an experimental model of neurodegeneration and hippocampal injury [4]. Altered intracellular Ca^2+^ homeostasis is considered a major mechanism underlying TMT-induced neurotoxicity [5]. TMT has been reported to increase intracellular Ca^2+^ influx, induce mitochondrial dysfunction, and activate inflammatory signaling pathways in neuronal cells [6]. However, most previous studies have focused primarily on acute neuronal injury, whereas relatively little is known about chronic TMT-induced Ca^2+^ dysregulation in glial and vascular-related cells. To address this issue, we investigated store-operated Ca^2+^ entry (SOCE) alterations in neuronal, astrocytic, microglial, brain endothelial, and vascular smooth muscle cells under chronic TMT exposure.

Ginsenoside compound K (CK), a bioactive metabolite of protopanaxadiol-type ginsenosides, exhibits anti-inflammatory, antioxidant, and neuroprotective effects [7]. Recent studies have demonstrated that CK alleviates brain aging by inhibiting ferroptosis through modulation of the ASK1-MKK7-JNK signaling pathway and enhancements in Nrf2-mediated antioxidant responses [7]. CK has also been shown to reduce neuronal damage and synaptic dysfunction by directly targeting amyloid-β in Alzheimer’s disease models [8] and to attenuate cerebral ischemia–reperfusion injury and PANoptosis-associated neuronal damage under neuroinflammatory conditions [9]. Because intracellular Ca^2+^ dysregulation is increasingly recognized as a core feature of neurodegenerative disease, restoration of pathological Ca^2+^ imbalance may represent a promising therapeutic strategy. However, whether CK modulates pathological SOCE dysregulation and intracellular Ca^2+^ homeostasis under neurotoxic stress conditions remains unclear. Because pathological Ca^2+^ remodeling is increasingly recognized as a potential pharmacological target in neurodegenerative disorders, identification of compounds capable of differentially modulating intracellular Ca^2+^ homeostasis across neuronal and neurovascular cell types may provide important therapeutic insight. Therefore, this study aimed to investigate the effects of CK on intracellular Ca^2+^ homeostasis and associated signaling pathways under chronic neurotoxic stress conditions.

Store-operated Ca^2+^ entry (SOCE) is a major mechanism regulating intracellular Ca^2+^ influx and cellular signaling. Depletion of endoplasmic reticulum (ER) Ca^2+^ stores activates SOCE through STIM/Orai- and transient receptor potential (TRP)-associated pathways [10]. To investigate signaling pathways associated with the effects of CK on SOCE, L-type Ca^2+^ channels (LTCCs), phospholipase C (PLC), and phospholipase D (PLD) were selected based on their reported involvement in neurodegenerative pathology. LTCC-associated Ca^2+^ signaling has been implicated in neuronal aging, oxidative stress responses, and excitotoxic neuronal injury [11]. In addition, ER Ca^2+^ depletion may activate TRP-associated signaling, contributing to membrane depolarization and secondary activation of LTCCs [12]. PLC-mediated signaling is also closely associated with aberrant ER Ca^2+^ release and altered intracellular Ca^2+^ dynamics in neurodegenerative disease models [13]. Furthermore, PLD signaling has been implicated in membrane remodeling, oxidative stress responses, and neurodegenerative disease progression [14]. These findings suggest that distinct Ca^2+^ signaling pathways may contribute to cell type-specific SOCE dysregulation under neurotoxic stress conditions.

Here, we investigated TMT-induced alterations of SOCE across multiple neuronal and vascular-related cell types and examined whether CK restores pathological Ca^2+^ dysregulation under chronic neurotoxic stress conditions. We further investigated pharmacological signaling pathways involved in CK-mediated Ca^2+^ regulation, focusing on PKA-LTCC-associated signaling in neuronal cells and PLC/PLD-associated signaling in astrocytes.

## 2. Results

### 2.1. Trimethyltin Chloride Induces Cell Type-Specific Ca^2+^ Dysregulation and Cellular Stress Responses

**Figure 1:**
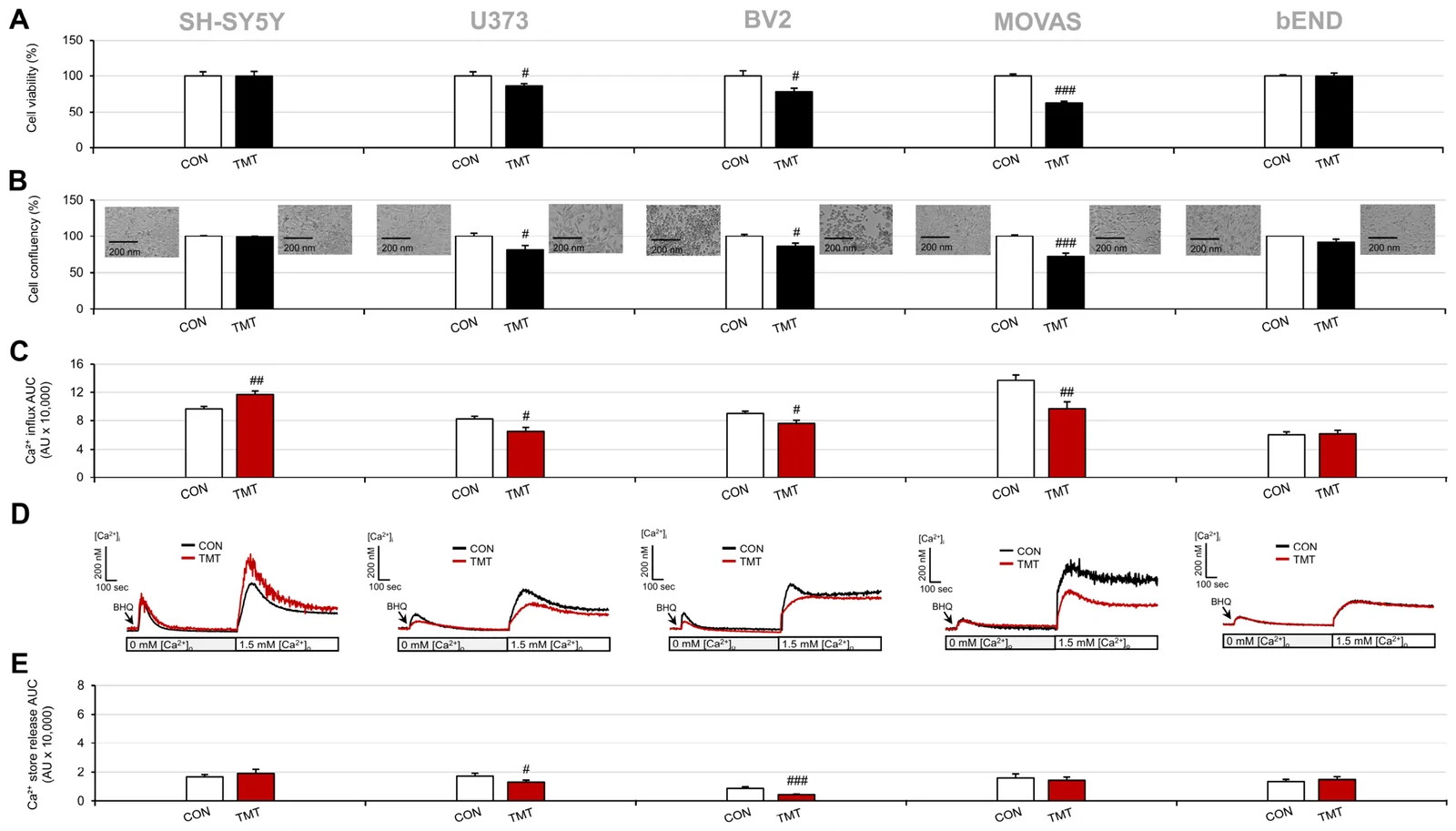
Cell type-specific cellular stress responses and Ca^2+^ dysregulation induced by TMT in SH-SY5Y, U373, BV2, bEND, and MOVAS cells. (**A**) Cell viability assessed by MTT activity (*n* = 11–16). (**B**) Representative images and quantitative analysis of cell confluence (*n* = 9–12). (**C**) Quantitative analysis of SOCE-mediated Ca^2+^ influx expressed as the area under the curve (AUC) for 500 s following re-addition of 1.5 mM extracellular Ca^2+^ (*n* = 12–13). (**D**) Representative intracellular Ca^2+^ traces obtained during SOCE measurements. (**E**) Quantitative analysis of ER Ca^2+^ store release expressed as the AUC following stimulation with 30 μM BHQ (*n* = 12–13). Data are presented as the mean ± SEM. Statistical analyses were performed using Student’s *t*-test. ^#^ *p* < 0.05, ^##^ *p* < 0.01, ^###^ *p* < 0.001 vs. control (CON). TMT, trimethyltin chloride; BHQ, 2,5-di-tert-butylhydroquinone; SOCE, store-operated Ca^2+^ entry.

The cytotoxic stress induced by TMT (5 μM, 24 h) was evaluated using cell viability and cell confluence analyses. TMT significantly reduced cell viability to 86.4%, 77.6%, and 62.3% in U373, BV2, and MOVAS cells, respectively (*p* = 0.041, *p* = 0.017, and *p* < 0.001; Figure 1A). Similarly, cell confluence was significantly decreased to 81.3%, 86.0%, and 72.3% in U373, BV2, and MOVAS cells, respectively (*p* = 0.022, *p* = 0.012, and *p* < 0.001; Figure 1B). In contrast, SH-SY5Y and bEND cells showed no significant changes in either cell viability or cell confluence following TMT exposure (Figure 1A,B).

We next investigated whether TMT altered intracellular Ca^2+^ dynamics in SH-SY5Y, U373, BV2, bEND, and MOVAS cells. TMT significantly increased SOCE-mediated Ca^2+^ influx in SH-SY5Y cells, whereas Ca^2+^ influx was significantly reduced in U373, BV2, and MOVAS cells, with the greatest reduction observed in MOVAS cells (*p* = 0.002, *p* = 0.013, *p* = 0.018, and *p* = 0.004, respectively; Figure 1C,D). In contrast, bEND cells exhibited minimal changes in Ca^2+^ influx following TMT exposure (Figure 1C,D). TMT also significantly reduced ER Ca^2+^ store release in U373 and BV2 cells (*p* = 0.049 and *p* < 0.001, respectively; Figure 1D,E). These findings demonstrate that identical TMT exposure induces distinct patterns of intracellular Ca^2+^ dysregulation and cellular injury across cell types. Notably, SH-SY5Y neuronal cells exhibited increased SOCE-mediated Ca^2+^ influx without overt cytotoxicity, indicating altered Ca^2+^ handling at the 24 h time point in the absence of detectable loss of cell viability.

### 2.2. Ginsenoside Compound K Restores TMT-Induced Ca^2+^ Dysregulation with Cell Type-Specific Pharmacological Profiles in SH-SY5Y and U373 Cells

**Figure 2:**
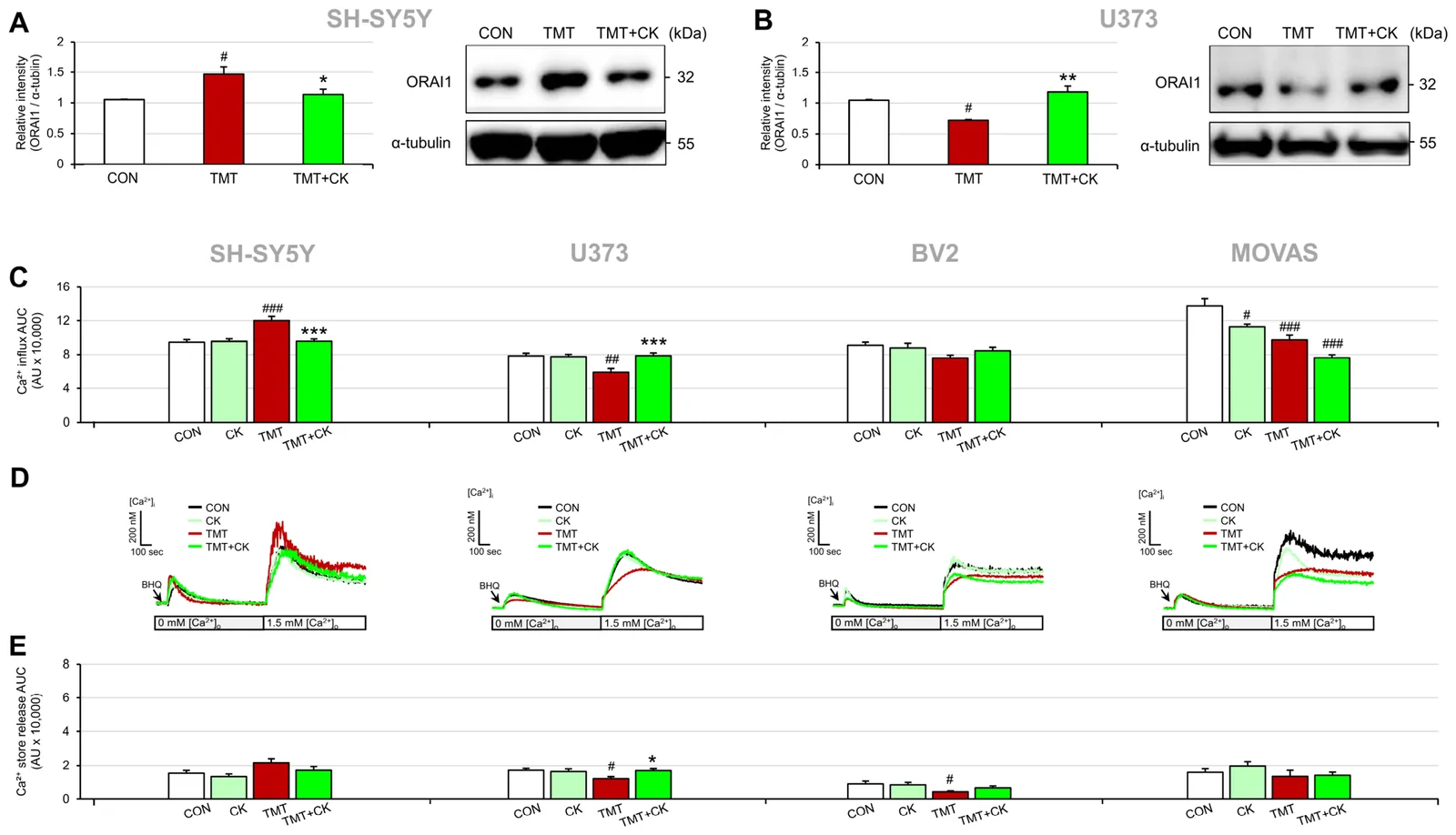
The effects of CK on Orai1 protein expression and TMT-induced Ca^2+^ dysregulation in SH-SY5Y, U373, BV2, and MOVAS cells. (**A**,**B**) Representative Western blots and quantitative analysis of Orai1 protein expression in SH-SY5Y and U373 cells following TMT (5 μM, 24 h) and CK (1 μM, 22 h) treatment (*n* = 3–5). (**C**) Quantitative analysis of SOCE-mediated Ca^2+^ influx expressed as the area under the curve (AUC) for 500 s following the re-addition of 1.5 mM extracellular Ca^2+^ in SH-SY5Y, U373, BV2, and MOVAS cells (*n* = 11–16). (**D**) Representative intracellular Ca^2+^ traces obtained during SOCE measurements. (**E**) Quantitative analysis of ER Ca^2+^ store release expressed as the AUC following stimulation with 30 μM BHQ (*n* = 11–16). Data are presented as the mean ± SEM. Statistical analyses were performed using one-way ANOVA followed by Tukey’s post hoc test. ^#^ *p* < 0.05, ^##^ *p* < 0.01, ^###^ *p* < 0.001 vs. control (CON); * *p* < 0.05, ** *p* < 0.01, *** *p* < 0.001 vs. TMT. CK, compound K; TMT, trimethyltin chloride; BHQ, 2,5-di-tert-butylhydroquinone; SOCE, store-operated Ca^2+^ entry.

To examine whether the functional SOCE alterations were accompanied by changes in Orai1 protein expression, we performed Western blot analysis of Orai1. In SH-SY5Y cells, TMT increased the Orai1 protein levels, whereas CK significantly attenuated the TMT-induced increase (*p* = 0.015 and *p* = 0.048; Figure 2A). In contrast, TMT decreased Orai1 protein levels in U373 cells, and CK restored Orai1 expression (*p* = 0.015 and *p* = 0.003; Figure 2B).

Treatment with CK alone did not significantly alter basal intracellular Ca^2+^ levels. However, CK significantly reversed TMT-induced Ca^2+^ dysregulation in both SH-SY5Y and U373 cells (*p* < 0.001 and *p* < 0.001, respectively; Figure 2C,D). Specifically, CK reduced the TMT-induced increase in Ca^2+^ influx in SH-SY5Y cells, whereas CK restored the TMT-induced decrease in Ca^2+^ influx in U373 cells. In addition, CK significantly restored the reduction in ER Ca^2+^ store release induced by TMT in U373 cells (*p* = 0.049; Figure 2D,E).

To investigate signaling pathways associated with CK-mediated Ca^2+^ regulation, pharmacological inhibitors or modulators of ROS-related signaling (NAC, 1 mM), TRP-associated signaling (ACA, 10 μM), SOCE (BTP2, 0.25 μM), PKA (H-89, 10 μM), LTCCs (nifedipine, 1 μM), PLD (FIPI, 500 nM), PLC (U73122, 1 μM), together with the inactive analog of U73122 (U73343, 1 μM), were examined.

**Figure 3:**
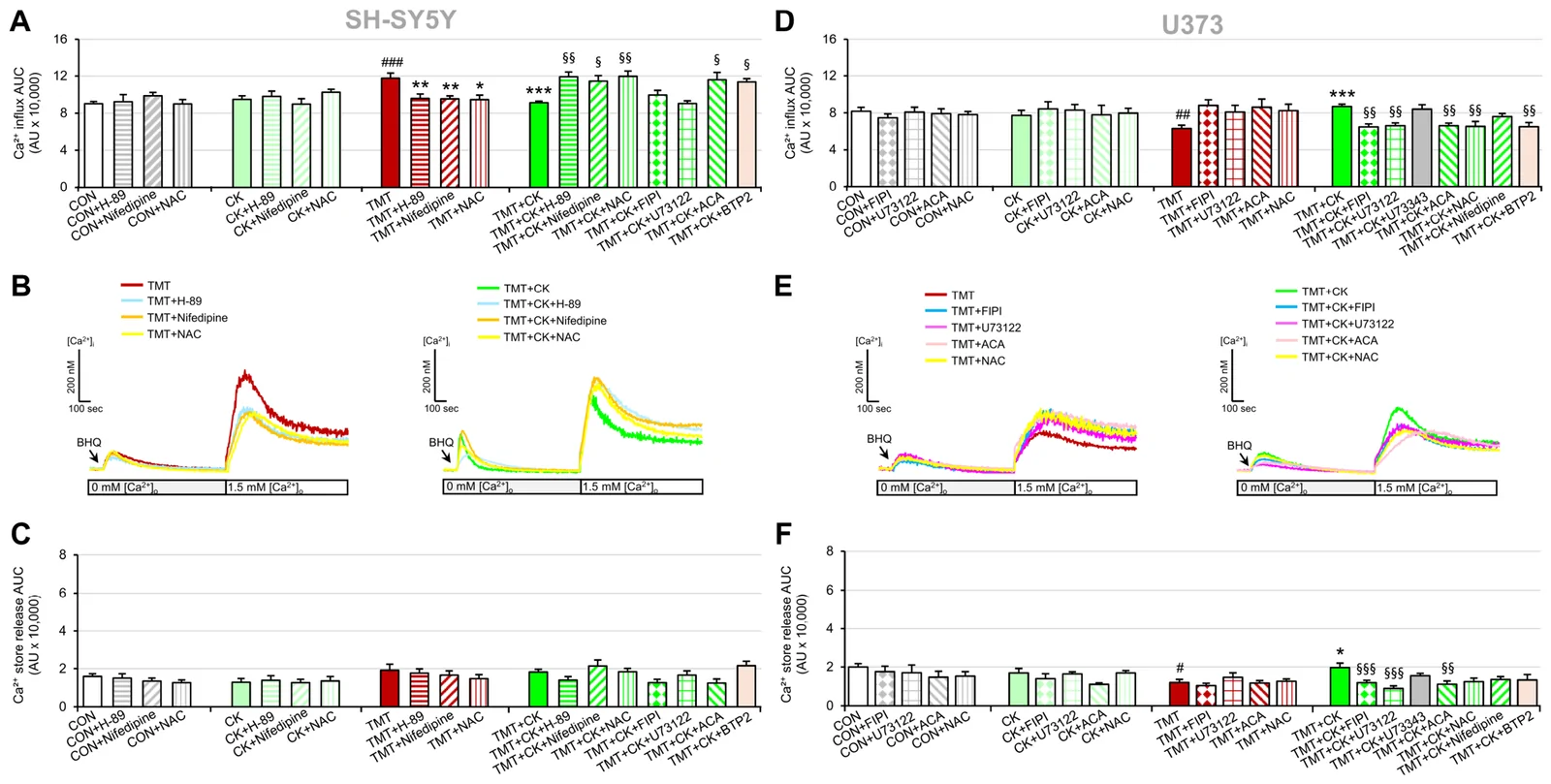
Pharmacological sensitivity profiles associated with CK-mediated regulation of intracellular Ca^2+^ homeostasis in SH-SY5Y and U373 cells. (**A**,**D**) Quantitative analysis of SOCE-mediated intracellular Ca^2+^ influx in control, CK-, TMT-, and TMT + CK-treated cells in the absence or presence of pharmacological inhibitors. (**B**,**E**) Representative intracellular Ca^2+^ traces showing ER Ca^2+^ store release induced by BHQ and subsequent SOCE-mediated Ca^2+^ influx after extracellular Ca^2+^ re-addition. (**C**,**F**) Quantitative analysis of ER Ca^2+^ store release following pharmacological inhibitor treatment. For quantitative analyses, *n* = 6–13 independent biological experiments were included, depending on the experimental group. Data are presented as the mean ± SEM. Statistical analyses were performed using one-way ANOVA followed by Tukey’s post hoc test. ^#^ *p* < 0.05, ^##^ *p* < 0.01, ^###^ *p* < 0.001 vs. control (CON); * *p* < 0.05, ** *p* < 0.01, *** *p* < 0.001 vs. TMT; ^§^ *p* < 0.05, ^§§^ *p* < 0.01, ^§§§^ *p* < 0.001 vs. TMT + CK. H-89, protein kinase A inhibitor; nifedipine, L-type Ca^2+^ channel blocker; NAC, N-acetyl-L-cysteine; ACA, N-(p-amylcinnamoyl)anthranilic acid; BTP2, SOCE inhibitor; FIPI, phospholipase D inhibitor; U73122, phospholipase C inhibitor; U73343, inactive analog of U73122; BHQ, 2,5-di-tert-butylhydroquinone; SOCE, store-operated Ca^2+^ entry; CK, compound K; TMT, trimethyltin chloride.

NAC, ACA, and BTP2 significantly attenuated the effects of CK in both SH-SY5Y cells (*p* = 0.005, *p* = 0.019, and *p* = 0.049, respectively; Figure 3A,B) and U373 cells (*p* = 0.003, *p* = 0.003, and *p* = 0.003, respectively; Figure 3D,E), indicating that CK-associated Ca^2+^ responses were pharmacologically sensitive to NAC, ACA, and BTP2 in both cell types.

In SH-SY5Y cells, the effects of CK were additionally attenuated by H-89 and nifedi-pine (*p* = 0.008 and *p* = 0.037, respectively; Figure 3A,B), consistent with the involvement of PKA- and LTCC-associated signaling. In contrast, inhibition of PLC and PLD significantly attenuated the effects of CK in U373 cells (*p* = 0.003 and *p* = 0.003, respectively; Figure 3D,E), whereas U73343 did not significantly affect the effects of CK (Figure 3D,E). Furthermore, restoration of ER Ca^2+^ store release by CK in U373 cells was significantly reduced by U73122 (*p* < 0.001; Figure 3E,F). U73343, an inactive analog of U73122, had no significant effect on CK-induced Ca^2+^ regulation, providing additional pharmacological support for a PLC-associated contribution to CK-mediated Ca^2+^ regulation in U373 cells (Figure 3D–F).

To further assess potential nonspecific pharmacological effects and strengthen the interpretation of CK-associated Ca^2+^ regulation, we examined the principal pathway-directed inhibitors under control, CK, and TMT conditions. None of the inhibitors significantly altered Ca^2+^ influx or Ca^2+^ release under control or CK conditions in SH-SY5Y cells (NAC, H-89, nifedipine; Figure 3A–C) or U373 cells (NAC, ACA, FIPI, U73122; Figure 3D–F) compared with their respective vehicle-treated groups. Under TMT treatment, the TMT-induced Ca^2+^ increase in SH-SY5Y cells was significantly reversed by these inhibitors, whereas the TMT-induced reduction in Ca^2+^ influx in U373 cells was partially reversed but did not reach statistical significance. Furthermore, under TMT + CK conditions, these inhibitors significantly attenuated the CK-associated normalization of Ca^2+^ homeostasis, whereas they produced no significant effects in the absence of TMT. These findings indicate that the inhibitors did not significantly perturb baseline Ca^2+^ handling under control or CK conditions and strengthen the interpretation of the observed cell type-specific pharmacological sensitivity profiles under TMT exposure.

**Figure 4:**
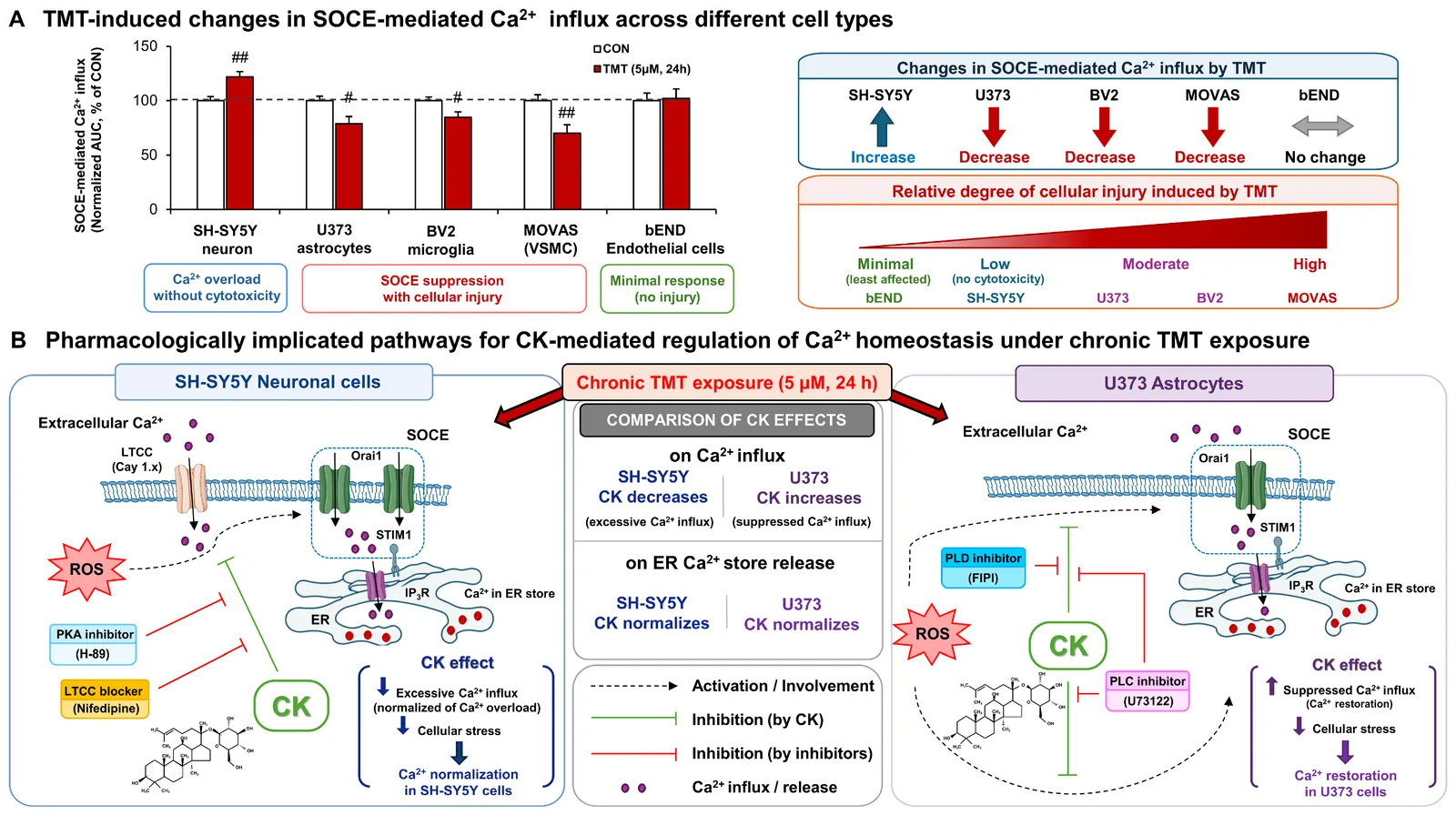
Cell type-specific TMT-induced alterations in SOCE-mediated Ca^2+^ influx and the pharmacologically implicated signaling profiles associated with CK-mediated Ca^2+^ regulation. (**A**) TMT-induced changes in SOCE-mediated Ca^2+^ influx across five cell types. Left: SOCE-mediated Ca^2+^ influx (normalized AUC, % of control) in SH-SY5Y, U373, BV2, MOVAS, and bEND cells following chronic TMT exposure (5 μM, 24 h) (*n* = 12–13). Data are presented as the mean ± SEM. Statistical analyses were performed using Student’s *t*-test. ^#^ *p* < 0.05, ^##^ *p* < 0.01 vs. control (CON). Right: Summary of TMT-induced changes in SOCE-mediated Ca^2+^ influx and the relative degree of cellular injury observed across cell types. SH-SY5Y cells exhibited increased SOCE-mediated Ca^2+^ influx without significant cytotoxicity, whereas U373, BV2, and MOVAS cells showed reduced SOCE-mediated Ca^2+^ influx accompanied by cellular injury. In contrast, bEND endothelial cells exhibited minimal alterations in intracellular Ca^2+^ influx and cellular stress following TMT exposure. (**B**) Summary of pharmacologically implicated signaling associated with CK-mediated Ca^2+^ regulation under TMT exposure in SH-SY5Y and U373 cells. Top center: Chronic TMT exposure (5 μM, 24 h) induced differential SOCE-mediated Ca^2+^ responses in neuronal and astrocytic cells. Left: In SH-SY5Y cells, CK-associated attenuation of TMT-induced Ca^2+^ influx was sensitive to H-89 and nifedipine, supporting the involvement of PKA- and LTCC-associated signaling. Right: In U373 cells, CK-associated restoration of suppressed Ca^2+^ influx was sensitive to U73122 and FIPI, supporting the involvement of PLC- and PLD-associated signaling. Green arrows indicate CK-associated modulation or restoration of Ca^2+^ responses; red inhibitory symbols indicate pharmacological inhibition; black dashed arrows indicate putative pathway relationships inferred from the pharmacological analyses. CK, compound K; TMT, trimethyltin chloride; LTCC, L-type Ca^2+^ channel; PLC, phospholipase C; PLD, phospholipase D; PKA, protein kinase A; ROS, reactive oxygen species; SOCE, store-operated Ca^2+^ entry; STIM1, stromal interaction molecule 1; Orai1, ORAI calcium release-activated calcium modulator 1; IP_3_R, inositol 1,4,5-trisphosphate receptor.

Collectively, these results indicate that CK differentially modulates intracellular Ca^2+^ homeostasis with both shared and cell type-specific pharmacological response profiles. In SH-SY5Y cells, CK-associated Ca^2+^ regulation was sensitive to H-89 and nifedipine, whereas in U373 cells, the CK-associated response was sensitive to U73122 and FIPI. Changes in Orai1 protein expression accompanied the corresponding alterations in SOCE-mediated Ca^2+^ influx in both cell types. A summary of the cell type-specific TMT responses and pharmacologically implicated CK-associated signaling profiles is presented in Figure 4.

## 3. Discussion

In this study, we demonstrated that chronic TMT exposure induces distinct patterns of SOCE dysregulation depending on cell type, highlighting a previously underrecognized aspect of TMT-induced Ca^2+^ pathology. SH-SY5Y neuronal cells exhibited increased SOCE-mediated Ca^2+^ influx without overt cytotoxicity, whereas astrocytes, microglia, and vascular smooth muscle cells showed reduced Ca^2+^ influx accompanied by cellular damage. In contrast, brain endothelial cells maintained both cellular viability and Ca^2+^ homeostasis following TMT exposure. Importantly, CK-associated restoration of abnormal Ca^2+^ responses showed distinct pharmacological sensitivity profiles depending on cell type. These findings suggest that therapeutic strategies targeting intracellular Ca^2+^ homeostasis in neurodegenerative disease may require a cell type-specific approach.

In neuronal cells, the increase in Ca^2+^ influx occurred in the absence of detectable loss of viability at the 24 h time point, indicating a dissociation between altered Ca^2+^ handling and overt cytotoxicity under the present experimental conditions. Previous studies have shown that oxidative stress and mitochondrial dysfunction induced by neurotoxic stimuli can activate PKA-associated signaling and subsequently enhance LTCC activity in neurons [15]. In the present study, CK attenuated TMT-induced Ca^2+^ influx, and the CK-associated reduction in Ca^2+^ influx was attenuated by H-89 and nifedipine. These pharmacological findings are consistent with involvement of PKA- and LTCC-associated signaling in CK-mediated Ca^2+^ regulation. Thus, the present data support pharmacological involvement of these pathways but do not establish their direct causal sequence. TMT-induced oxidative stress may contribute to altered LTCC-associated Ca^2+^ signaling in neuronal cells, thereby contributing to the observed increase in Ca^2+^ influx.

Astrocytes and microglial cells showed reduced Ca^2+^ influx and ER Ca^2+^ store release following chronic TMT exposure. This pattern differs markedly from that observed in neuronal cells and may reflect altered ER Ca^2+^ handling and SOCE regulation under sustained neurotoxic stress conditions. Previous studies have suggested that chronic oxidative stress and mitochondrial dysfunction may suppress SOCE through ER Ca^2+^ depletion and impaired store-operated signaling [16]. Although STIM–Orai signaling is a central component of SOCE, its functional coupling was not directly assessed in the present study. Compared with neuronal cells, astrocytes and microglia exhibited a distinct response pattern characterized by reduced SOCE and greater cellular injury; however, the temporal relationship among these responses cannot be determined from the single 24 h time point examined in this study.

In astrocytes, CK-associated restoration in both Ca^2+^ influx and ER Ca^2+^ store release was sensitive to PLC and PLD inhibition. PLD signaling is also closely associated with membrane dynamics, oxidative stress responses, and pathological cellular remodeling under neurodegenerative and neoplastic conditions [14]. In addition, dysregulated intracellular Ca^2+^ signaling and PLD activity have been implicated in cancer progression and abnormal cell proliferation [17]. Therefore, the present findings, together with previous evidence showing that CK suppresses prostate cancer cell cycle progression [18], raise the possibility that PLD-associated Ca^2+^ regulation may contribute to the broader biological effects of CK, although this possibility was not directly examined in the present study.

Although microglial cells exhibited reduced SOCE similar to astrocytes, CK showed only limited restorative effects. This reduced responsiveness may be associated with the greater degree of cellular injury observed in microglia under the present experimental conditions. Similarly, vascular smooth muscle cells exhibited the greatest reduction in both cell viability and intracellular Ca^2+^ influx, and CK failed to restore Ca^2+^ homeostasis in these cells. These findings suggest that the efficacy of CK may depend on the degree of cellular injury and residual Ca^2+^ regulatory capacity. Notably, MOVAS vascular smooth muscle cells showed the greatest reduction in both intracellular Ca^2+^ influx and cellular viability following identical TMT exposure, suggesting particularly high vulnerability of vascular smooth muscle cells to chronic neurotoxic stress. Because cerebrovascular dysfunction and vascular stiffness are increasingly recognized as important contributors to cognitive decline and AD progression [19], the pronounced Ca^2+^ and viability changes observed in MOVAS cells warrant further investigation of vascular Ca^2+^ dysregulation in neurodegenerative models.

Notably, brain endothelial cells maintained both cellular viability and intracellular Ca^2+^ homeostasis following chronic TMT exposure. Unlike neuronal, astrocytic, microglial, and vascular smooth muscle cells, bEND cells exhibited minimal alterations in SOCE-mediated Ca^2+^ influx, suggesting relative resistance to TMT-induced neurotoxic stress. This differential response may reflect distinct Ca^2+^ signaling architecture in brain endothelial cells. Previous studies have suggested that endothelial SOCE may depend predominantly on TRPC1- and TRPC4-associated pathways, which may provide compensatory Ca^2+^ handling capacity and resistance to oxidative stress-associated Ca^2+^ dysregulation [20,21,22]. However, whether these pathways account for the relative stability observed in bEND cells was not directly examined in the present study. In addition, our previous findings demonstrated that bEND cells respond differently to oxidative stress compared with neuronal and glial cell types, supporting the possibility that endothelial cells possess distinct protective mechanisms against chronic neurotoxic injury [20]. Taken together, these findings suggest that brain endothelial cells may maintain intracellular Ca^2+^ stability through distinct SOCE-associated regulatory mechanisms, although the specific molecular basis requires further investigation.

CK was administered to cell types exhibiting significant SOCE alterations following TMT exposure, including neuronal cells, astrocytes, microglia, and vascular smooth muscle cells. To model a post-exposure intervention paradigm, CK treatment was initiated 2 h after TMT exposure rather than as a pretreatment paradigm. To our knowledge, this study provides evidence that CK is associated with modulation of intracellular Ca^2+^ homeostasis under chronic neurotoxic stress conditions, extending its previously reported antioxidant and anti-inflammatory properties to regulation of pathological Ca^2+^ signaling [7]. Importantly, CK normalized intracellular Ca^2+^ responses bidirectionally, attenuating excessive Ca^2+^ influx in neuronal cells while restoring suppressed Ca^2+^ signaling in astrocytes. These findings suggest that CK may function as a Ca^2+^ homeostatic regulator rather than as a simple Ca^2+^ inhibitor. At the molecular level, Orai1 protein expression closely paralleled the functional SOCE responses in both SH-SY5Y and U373 cells. TMT significantly increased Orai1 expression in SH-SY5Y cells, whereas CK attenuated this increase. In contrast, TMT reduced Orai1 expression in U373 astrocytes, and CK restored Orai1 expression toward control levels. These molecular findings are consistent with the Ca^2+^ imaging data and suggest that CK-mediated regulation of intracellular Ca^2+^ homeostasis is associated with cell type-specific changes in Orai1 expression accompanying the functional SOCE responses. Previous studies have demonstrated that impaired STIM1/Orai1-associated SOCE contributes to astrocytic Ca^2+^ hypoactivity and synaptic dysfunction in Alzheimer’s disease models, whereas restoration of STIM1-dependent SOCE improves astrocytic function and neuronal plasticity [16,23]. Importantly, the additional pharmacological control experiments showed no significant effects under control or CK-only conditions, strengthening the pharmacological interpretation of the inhibitor effects under TMT + CK conditions. Responses in both cell types showed pharmacological sensitivity to NAC, ACA, and BTP2, suggesting partially overlapping pharmacological response profiles. Collectively, these findings support the possibility that CK is associated with restoration of intracellular Ca^2+^ balance under conditions of TMT-induced SOCE dysregulation.

Our findings demonstrate that identical TMT exposure produces distinct patterns of SOCE dysregulation and cellular injury depending on cell type. Overt cellular damage was observed predominantly in cells exhibiting reduced Ca^2+^ influx, whereas SH-SY5Y cells showed increased SOCE-mediated Ca^2+^ influx without significant loss of viability at 24 h. These observations indicate cell type-specific differences in Ca^2+^ regulation and vulnerability under the present experimental conditions; however, they do not establish a temporal sequence of neurotoxic injury.

The present results further support investigation of cell type-specific Ca^2+^ homeostasis as a potential therapeutic target in neurodegenerative disorders. However, because only a single TMT exposure time was examined, the present findings should not be interpreted as defining early or late temporal stages of Ca^2+^ dysregulation. Accordingly, cell type-specific restoration of intracellular Ca^2+^ balance warrants further investigation as a therapeutic concept in neurodegenerative models. Realization of such strategies will require technologies capable of monitoring intracellular Ca^2+^ dynamics with cell type resolution. Recent experimental evidence has shown that astrocyte-specific Ca^2+^ modulation enhances neurological recovery following cerebral infarction, further supporting the therapeutic relevance of cell type-specific Ca^2+^ regulation in neurological disorders [24].

This study has several limitations. First, individual cell types were examined separately rather than in co-culture systems, limiting evaluation of intercellular interactions within the neurovascular unit. Second, although pharmacological inhibition studies suggested involvement of specific signaling pathways, the precise molecular mechanisms underlying CK-mediated Ca^2+^ regulation remain incompletely understood. Therefore, additional mechanistic studies are required to further clarify the molecular basis of CK-mediated Ca^2+^ homeostasis under neurotoxic stress conditions. Third, only a single CK concentration (1 μM) was examined, and a formal dose–response analysis was not performed. Therefore, the optimal concentration range and concentration dependence of CK-mediated Ca^2+^ regulation remain to be established. Fourth, TMT-induced responses were evaluated at a single 24 h time point; therefore, temporal progression or early-versus-late stages of Ca^2+^ dysregulation cannot be inferred from the present data. Fifth, the cell lines were not independently re-authenticated after acquisition, and routine mycoplasma testing was not performed. These methodological limitations should be considered when interpreting the findings.

## 4. Materials and Methods

### 4.1. Cell Culture

Human neuroblastoma SH-SY5Y, astrocytic U373, microglial BV2, brain endothelial bEND, and vascular smooth muscle MOVAS cells were obtained from the American Type Culture Collection (ATCC; Manassas, VA, USA) and maintained in Dulbecco’s Modified Eagle Medium (DMEM) supplemented with 10% fetal bovine serum and 1% penicillin/streptomycin at 37 °C in a humidified atmosphere containing 5% CO_2_. No additional in-house cell line authentication was performed after acquisition. Cells between passages 10 and 20 were used for the experiments. Routine mycoplasma testing was not performed during the study. Cell cultures were maintained under aseptic conditions and routinely monitored microscopically for gross morphological abnormalities. For experimental assays, cells were seeded at 1 × 10^4^ cells/well in 96-well plate for the MTT assay, 2 × 10^4^ cells/well in 24-well plate for real-time cell confluence analysis, and 1 × 10^6^ cells/dish in a 60 mm dish for Western blot analysis. For intracellular Ca^2+^ measurements, cells were prepared at 1 × 10^6^ cells/mL, as described below.

To induce neurotoxic stress conditions, cells were exposed to trimethyltin chloride (TMT, 5 μM) for 24 h, as previously described [25]. For evaluation of the effects of ginsenoside compound K (CK) on intracellular Ca^2+^ regulation, cells were treated with CK (1 μM) for 22 h beginning 2 h after TMT exposure. CK (1 μM) was selected as an exploratory low-micromolar working concentration for the present study. Because a formal dose–response analysis was not performed, this concentration should not be interpreted as an optimized dose.

### 4.2. Cell Viability

Cell viability was evaluated using the MTT assay. Cells were treated with TMT (5 μM) for 24 h with or without CK (1 μM) using the same treatment sequence as in Ca^2+^ imaging experiments. Cells were seeded in 96-well plates and incubated with MTT solution (0.5 mg/mL) for 1 h following treatment. Absorbance was measured at 570 and 690 nm using a microplate reader (SPECTROstar Nano 4.2; BMG LABTECH, Ortenberg, Germany). Cell viability was expressed as a percentage relative to the vehicle-treated control group.

### 4.3. Real-Time Cell Confluence Analysis

To further evaluate TMT-induced cytotoxic stress and proliferation changes, cells were allocated to vehicle-treated control or TMT-treated groups. Real-time cell confluence was monitored using an automated live-cell imaging system (Celloger Mini Plus; Curiosis Inc., Seoul, Republic of Korea). Confluence values were continuously recorded for 24 h after treatment, and changes in proliferation dynamics were analyzed.

### 4.4. Measurement of Intracellular Ca^2+^

Intracellular Ca^2+^ dynamics were evaluated using Fura-2-based ratiometric fluorescence analysis. Cells were suspended in extracellular solution containing 150 mM NaCl, 10 mM HEPES, 10 mM glucose, 6 mM KCl, 1.5 mM CaCl_2_, and 1 mM MgCl_2_ (pH 7.4 adjusted with NaOH). Cells (1 × 10^6^ cells/mL) were loaded with Fura-2 acetoxymethyl ester (Fura-2 AM, 2.5 μM) for 30 min at room temperature. SOCE was induced by depletion of endoplasmic reticulum (ER) Ca^2+^ stores using 30 μM 2′,5′-di(tert-butyl)-1,4-benzohydroquinone (BHQ), followed by re-addition of 1.5 mM extracellular Ca^2+^, as previously described [26]. Fluorescence measurements were performed using a spectrofluorometer (Photon Technology Ltd., Edison, NJ, USA) with excitation wavelengths of 340 and 380 nm and emission at 510 nm. Quantitative analyses were performed using Microcal Origin 6.0 software (Microcal software Inc., Northampton, MA, USA).

To investigate signaling pathways associated with CK-mediated Ca^2+^ regulation, pharmacological inhibitors targeting transient receptor potential-associated signaling (ACA, 10 μM), reactive oxygen species (ROS; N-acetyl-L-cysteine, 1 mM), SOCE (BTP2, 0.25 μM), LTCCs (nifedipine, 1 μM), PLD (FIPI, 500 nM), PLC (U73122, 1 μM), inactive analog of U73122 (U73343, 1 μM), and protein kinase A (PKA; H-89, 10 μM) were used.

### 4.5. Western Blotting Analysis

To evaluate changes in Orai1 protein expression accompanying the observed SOCE alterations, Orai1 protein expression was analyzed by Western Blot in SH-SY5Y and U373 cells. SH-SY5Y and U373 cells were exposed to TMT (5 μM, 24 h) and CK (1 μM, 22 h) following the same treatment protocol used for the SOCE experiments. After treatment, cells were washed with ice-cold phosphate-buffered saline (PBS) and lysed in PRO-PREP Protein Extraction Solution (Cat. No. 17081; iNtRON Biotechnology, Seongnam, Republic of Korea) according to the manufacturer’s instructions. Cell lysates were centrifuged at 12,000× *g* for 30 min at 4 °C, and the supernatants were collected. Protein concentrations were determined using a Pierce^™^ BCA Protein Assay Kit (Cat. No. 23225; Thermo Fisher Scientific, Rockford, IL, USA) according to the manufacturer’s instructions. Equal amounts of protein (30 μg per lane) were separated by 10% sodium dodecyl sulfate–polyacrylamide gel electrophoresis (SDS-PAGE) and transferred onto nitrocellulose membranes. Membranes were blocked with 5% BSA in Tris-buffered saline containing 0.1% Tween-20 (TBST) for 1 h at room temperature and then incubated overnight at 4 °C with primary antibodies against Orai1 (Proteintech, Cat. No. 66223-1-Ig; 1:5000) and α-tubulin (Abclon, Cat. No. AbC-2001; 1:2000).

After washing with TBST, membranes were incubated with the appropriate horseradish peroxidase-conjugated secondary antibodies (goat anti-mouse IgG for Orai1 and goat anti-rabbit IgG for α-tubulin; GeneTex, Cat. Nos. GTX2113111-01 and GTX2113110-01; 1:2000) for 1 h at room temperature. Immunoreactive bands were detected using an enhanced chemiluminescence (ECL) detection kit (Cat. No. AbC-3001; AbClon, Inc., Seoul, Republic of Korea). Protein bands were visualized using a Fujifilm Image Quant LAS-4000 Mini Luminescent Image Analyzer (Fujifilm, Minato-ku, Tokyo, Japan), and band intensities were quantified by densitometric analysis using ImageJ software (version 1.54p; National Institutes of Health, Bethesda, MD, USA). All protein expression levels were normalized to α-tubulin as a loading control.

### 4.6. Chemicals

Dulbecco’s Modified Eagle’s Medium (DMEM) and phosphate-buffered saline (PBS) were obtained from Welgene (Gyeongsan, Republic of Korea). Penicillin/streptomycin and fetal bovine serum (FBS) were purchased from Biowest (Riverside, MO, USA). MTT reagent was obtained from Amresco (Solon, OH, USA), and Fura-2 acetoxymethyl ester (Fura-2 AM; F1221, purity > 95%) was purchased from Invitrogen (Paisley, UK). Anti-Orai1 antibody (Proteintech, Cat. No. 66223-1-Ig, Rosemont, IL, USA) and anti-α-tubulin antibody (Abclon, Cat. No. AbC-2001, Seoul, Republic of Korea) were purchased from their respective manufacturers. All other chemicals, including TMT, CK, EGTA, BHQ, CaCl_2_, ionomycin, Triton X-100, dimethyl sulfoxide (DMSO), trypsin, nifedipine (Cat. No. N7634), H-89 (Cat. No. B1427), U73122 (Cat. No. U6881), U73343 (Cat. No. 662041), FIPI (Cat. No. 528245), N-(p-Amylcinnamoyl)anthranilic acid (ACA; Cat. No. A8486), N-acetyl-L-cysteine (NAC; Cat. No. A7250), and BTP2 (YM-58483; Cat. No. 203890-M), were purchased from Sigma-Aldrich (St. Louis, MO, USA). Fura-2 AM, BHQ, ionomycin, H-89, U73122, U73343, FIPI, BTP2, ACA, and CK were dissolved in DMSO, whereas all other reagents were dissolved in distilled water. The final concentration of DMSO in all experiments was maintained at ≤0.1%, and equivalent concentrations of DMSO were applied to control groups.

### 4.7. Statistical Analysis

Data are presented as the mean ± standard error of the mean (SEM). Statistical analyses were performed using IBM SPSS Statistics version 29 (IBM Corp., Armonk, NY, USA). Comparisons among multiple groups were analyzed using one-way analysis of variance (ANOVA) followed by Tukey’s post hoc test. Comparisons between two groups were performed using Student’s *t*-test. A *p*-value < 0.05 was considered statistically significant.

## 5. Conclusions

This study demonstrates that chronic TMT exposure induces distinct patterns of SOCE dysregulation depending on cell type, reflecting differential cellular vulnerability to neurotoxic stress. CK was associated with restoration of TMT-induced Ca^2+^ dysregulation with distinct pharmacological response profiles, attenuating elevated Ca^2+^ influx in neuronal cells while restoring suppressed Ca^2+^ signaling in astrocytes. These findings suggest that maintenance of cell type-specific Ca^2+^ homeostasis warrants further investigation as a therapeutic concept in neurodegenerative disease models, including AD. Furthermore, the present results highlight cell type-specific differences in Ca^2+^ responses and cellular vulnerability under neurotoxic stress conditions. Future development of technologies capable of monitoring cell type-specific intracellular Ca^2+^ dynamics may help clarify the translational relevance of cell type-specific Ca^2+^ regulation in neurodegenerative disorders.

## Data Availability

The raw data supporting the conclusions of this article will be made available by the authors on request.
